# Sub-Saharan Africa and Eurasia Ancestry of Reassortant Highly Pathogenic Avian Influenza A(H5N8) Virus, Europe, December 2019

**DOI:** 10.3201/eid2607.200165

**Published:** 2020-07

**Authors:** Edyta Świętoń, Alice Fusaro, Ismaila Shittu, Krzysztof Niemczuk, Bianca Zecchin, Tony Joannis, Francesco Bonfante, Krzysztof Śmietanka, Calogero Terregino

**Affiliations:** National Veterinary Research Institute, Puławy, Poland (E. Świętoń, K. Niemczuk, K. Śmietanka);; Istituto Zooprofilattico Sperimentale delle Venezie, Legnaro, Italy (A. Fusaro, B. Zecchin, F. Bonfante, C. Terregino);; National Veterinary Research Institute, Vom, Nigeria (I. Shittu, T. Joannis)

**Keywords:** highly pathogenic influenza A(H5N8) virus, influenza virus, viruses, subtype, influenza, ancestry, reassortant viruses, clade 2.3.4.4b, phylogeny, respiratory infections, Europe, sub-Saharan Africa, Eurasia

## Abstract

We report detection of a highly pathogenic avian influenza A(H5N8) clade 2.3.4.4b virus in Europe. This virus was generated by reassortment between H5N8 subtype virus from sub-Saharan Africa and low pathogenicity avian influenza viruses from Eurasia.

Highly pathogenic avian influenza (HPAI) H5 viruses belonging to clade 2.3.4.4 of the Goose/Guangdong/96 (GS/Gd) lineage continue to pose a threat to poultry and wild birds worldwide (*1**–**6*). Reassortment events between HPAI H5 and low pathogenicity avian influenza (LPAI) viruses of wild-bird origin have led to generation of novel variants that might be periodically spread by wild birds across continents (*6*).

After detection of the unofficially defined clade 2.3.4.4b (*7*) in May 2016 in Lake Uvs-Nur, Russia (*8*), and Qinghai Lake, China (*9*), the virus spread to Europe and Africa, causing one of the largest epizootics reported (*1*). This virus reached several countries in northern, western, eastern, central, and southern areas of Africa (*10*). Nigeria, Namibia, South Africa (*11*), and Egypt reported H5N8 cases throughout 2019, suggesting ongoing circulation of the virus in Africa.

No HPAI H5N8 viruses were detected in Europe during June–November 2019 (*12*). We report detection of a reassortant HPAI A(H5N8) clade 2.3.4.4b virus in Europe during December 2019.

## The Study

In July 2019, in the framework of active surveillance measures implemented in live bird markets in 18 of the 36 states in Nigeria, the National Veterinary Research Institute in Vom, Nigeria, identified a HPAI H5N8 virus in a guinea fowl in the southwestern state of Ogun. Months later, at the end of December 2019, a suspicion of an HPAI virus was raised in a holding of 14-week-old meat turkeys in Poland, located near water bodies (fish ponds and lakes of the Łęczna-Włodawa Lakeland). A sudden increase in deaths was observed, accompanied by neurologic signs such as trembling, inability to walk, paralysis of the wings, and pedaling movements of the legs. A total of 3,000–5,000 birds died during the first 3 days after the onset of clinical signs. Organ samples submitted to the National Reference Laboratory for Avian influenza at the National Veterinary Research Institute, Pulawy, Poland, were positive for avian influenza virus and were characterized as HPAI H5N8.

We conducted antigenic characterization of the virus isolate by using the hemagglutination inhibition (HI) assay, which showed that the H5N8 virus in Poland had higher antigenic reactivity with European Union Reference Laboratory reference HPAI H5N8 A/turkey/Italy/7898/2014 (IT-7898) chicken antiserum (clade 2.3.4.4, GS/Gd lineage) compared with reactivity determined for European Union Reference Laboratory HPAI H5N1 A/chicken/Scotland/1/59 (SCOT-59) and LPAI H5N3 A/teal/England/7394–2805/06 (ENG-7394) antiserum.

A comparison of HI titers obtained with the IT-7898, SCOT-59 and ENG-7394 homologous antigens and those recorded against the strain from Poland showed differences of 2 log2, 4 log2, and 5 log2, respectively. The H5N1 and H5N3 strains belong to the H5 Eurasian lineage and are unrelated to the GS/Gd lineage, which supports the marked difference in reactivity. Pathotyping of the virus from Poland by using the intravenous pathogenicity index recorded a value of 3.0, confirming the highly pathogenic phenotype in chickens.

As of January 31, 2020, a total of 20 outbreaks in poultry (commercial and backyard holdings) and 1 case in a wild bird (a dead goshawk found near the index farm) had been detected in different regions from eastern to western Poland. Since the reassortant virus was detected in Poland, the World Organisation for Animal Health has been notified about similar outbreaks in Slovakia, Hungary, Romania, Germany, and the Czech Republic. A greater white-fronted goose (*Anser albifrons*) in Germany, near the border with Poland, was also found to be infected (*13*).

**Figure 1:**
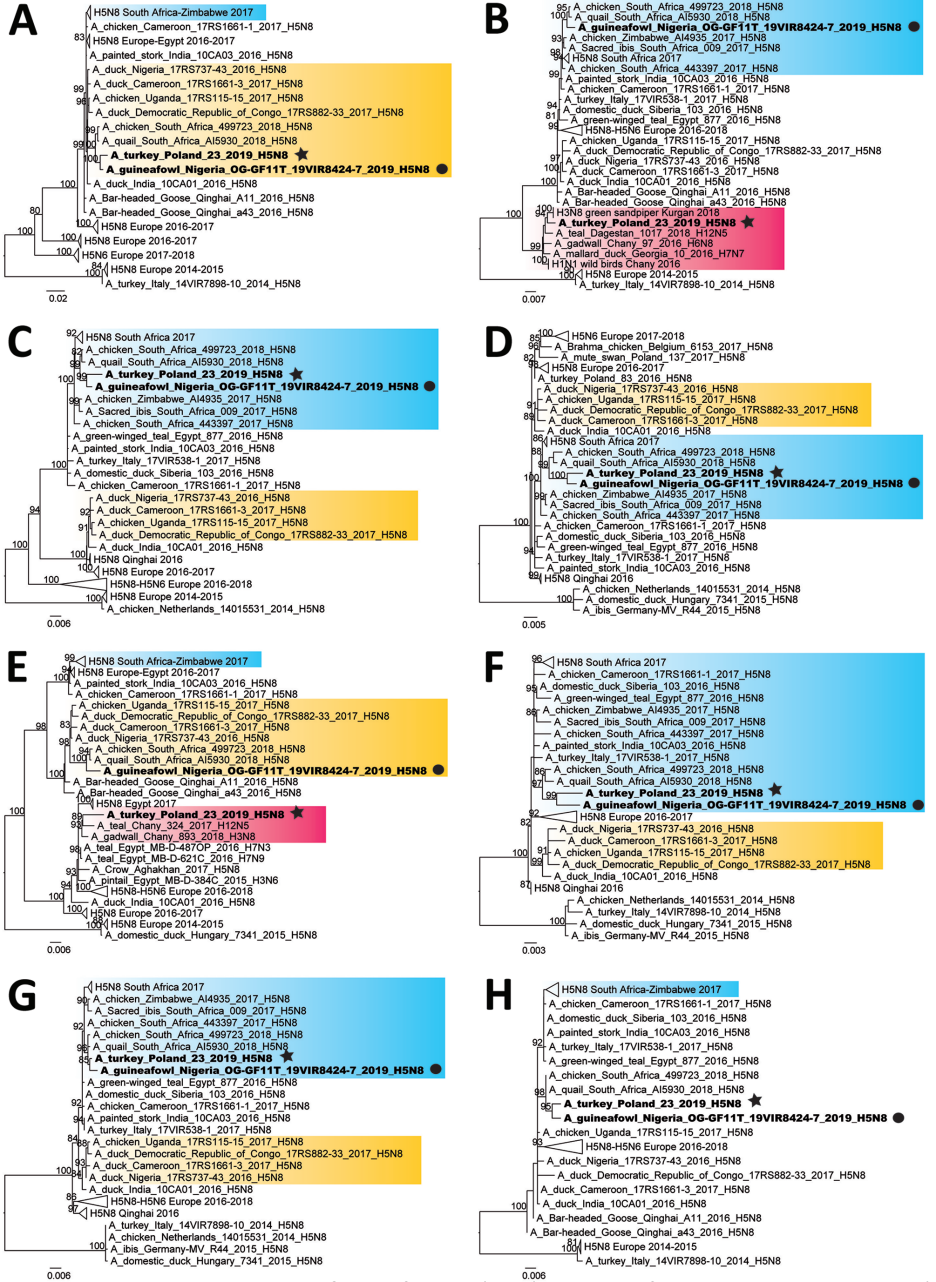
Maximum-likelihood phylogenetic trees of avian influenza A (H5N8) viruses identified in Poland and Nigeria, 2019. A) Polymerase basic protein 2, C) polymerase basic protein 1, C) polymerase acidic protein, D) hemagglutinin, E) nucleoprotein, F) neuraminidase, G) matrix protein, H) nonstructural protein. H5N8 viruses detected in Poland and Nigeria in 2019 are indicated in bold, A/turkey/Poland/23/2019(H5N8) is indicated by a black star, and A/guinea_fowl/Nigeria/OG-GF11T_19VIR8424–7/2019(H5N8) is indicated by a black circle. Blue box indicates the South Africa 2017 H5N8 cluster, yellow boxes indicate the West-Central Africa 2016–2017 cluster, and purple box indicates Eurasian LPAI viruses. Numbers next to each node represent ultrafast bootstrap supports (>80). Scale bars indicate nucleotide substitutions per site. LPAI, low pathogenicity avian influenza virus.

The genomes of HPAI H5N8 strains from index cases from Nigeria (A/guinea fowl/Nigeria/OG-GF11T_19VIR8424–7/2019) and Poland (A/turkey/Poland/23/2019) have been sequenced (Appendix) and submitted to the GISAID EpiFlu database (https://www.gisaid.org) under isolate nos. EPI_ISL_405278 and EPI_ISL_402134, respectively. Phylogenetic analysis (Appendix) of the hemagglutinin (HA) gene showed that both viruses belonged to clade 2.3.4.4b (Figure 1).

Topology of the polymerase basic protein 2, polymerase acidic protein, HA, neuraminidase, matrix, and nonstructural protein gene phylogenies showed that the viruses from Poland and Nigeria clustered together (nucleotide identity 98.9%–99.5%) (Table) and also with two 2018 H5N8 viruses from South Africa (*3*), which have been demonstrated to be a novel genotype that originated from reassortment events between clade 2.3.4.4b H5N8 viruses from South Africa and West-Central Africa (Figure 1). In contrast, the 2 strains clustered separately for the polymerase basic protein 1 and nucleoprotein gene phylogenies (Figure 1).

**Table:** Nucleotide identity of A/turkey/Poland/23/2019(H5N8) and A/guinea_fowl/Nigeria/OG-GF11T_19VIR8424–7/2019 influenza virus gene segments with the most similar sequences available in GenBank and EpiFlu*

Gene segment	Virus 1	Virus 2	Nucleotide identity, %
PB2	H5N8 Poland 2019†	H5N8 Nigeria 2019‡	98.9
	H5N8 Nigeria 2019	H5N8 South Africa 2018§	98.7
PB1	H5N8 Poland 2019	H3N8 Kurgan 2018¶	99.1
	H5N8 Poland 2019	H5N8 Nigeria 2019	94.7
	H5N8 Nigeria 2019	H5N8 South Africa 2018	99.2
PA	H5N8 Poland 2019	H5N8 Nigeria 2019	98.9
	H5N8 Nigeria 2019	H5N8 South Africa 2018	99.2
HA	H5N8 Poland 2019	H5N8 Nigeria 2019	99.1
	H5N8 Nigeria 2019	H5N8 South Africa 2018	98.7
NP	H5N8 Poland 2019	H3N8 Chany 2018#	98.5
	H5N8 Poland 2019	H5N8 Nigeria 2019	92.8
	H5N8 Nigeria 2019	H5N8 South Africa 2018	98.9
NA	H5N8 Poland 2019	H5N8 Nigeria 2019	98.8
	H5N8 Nigeria 2019	H5N8 South Africa 2018	98.9
M	H5N8 Poland 2019	H5N8 Nigeria 2019	99.5
	H5N8 Nigeria 2019	H5N8 South Africa 2018	99.4
NS	H5N8 Poland 2019	H5N8 Nigeria 2019	99.0
	H5N8 Nigeria 2019	H5N8 South Africa 2018	99.2

*HA, hemagglutinin; M, matrix protein; NA, neuraminidase; NP, nucleoprotein; NS, nonstructural protein; PA, polymerase acidic protein; PB1, polymerase basic protein 1; PB2, polymerase basic protein 2. †A/turkey/Poland/23/2019(H5N8).  ‡A/guinea_fowl/Nigeria/OG-GF11T_19VIR8424–7/2019(H5N8).  §A/chicken/South Africa/499723/2018(H5N8).  ¶A/green sandpiper/Kurgan/1043/2018(H3N8).  #A/gadwall/Chany/893/2018(H3N8).

**Figure 2:**
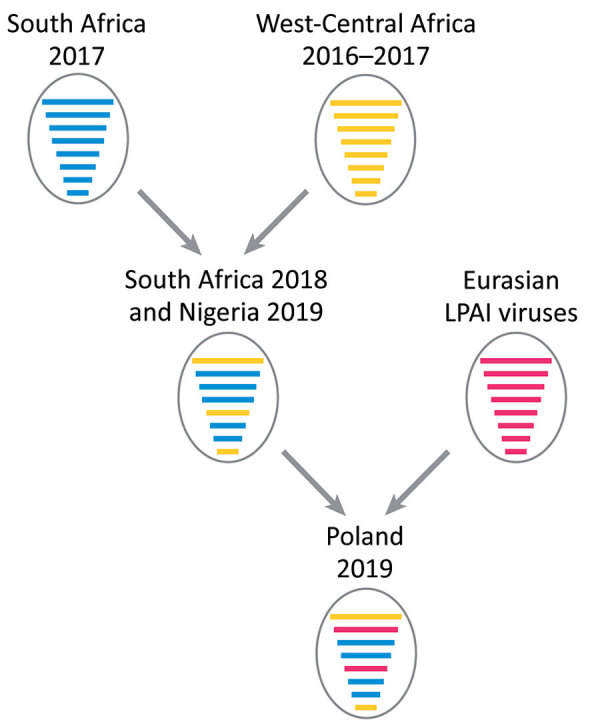
Reassortment events between highly pathogenic avian influenza virus H5N8 viruses from South Africa (2017), HPAI H5N8 viruses from West-Central Africa (2017); and LPAI viruses from Eurasia giving rise to A/guinea_fowl/Nigeria/OG-GF11T_19VIR8424–7/2019(H5N8) (Nigeria 2019) and A/turkey/Poland/23/2019(H5N8) (Poland 2019). Each gene segment is represented by a bar of different length, from top to bottom: polymerase basic protein 2 (PB2), polymerase basic protein 1 (PB1), polymerase acidic protein (PA), hemagglutinin (HA), nucleoprotein (NP), neuraminidase (NA), matrix protein (M), and nonstructural protein (NS). Each color represents a different viral origin: blue, H5N8 South Africa (2017); yellow, H5N8 West-Central Africa (2017); purple, Eurasian LPAI viruses. LPAI, low pathogenicity avian influenza virus.

For the polymerase basic protein 1 and nucleoprotein genes, the virus from Nigeria grouped with the 2018 viruses from South Africa as for the other gene segments, whereas the virus from Poland clustered with LPAI viruses identified in recent years in the Chany and Kurgan regions of Russia. This finding indicates that the strain from Poland is a reassortant virus derived from LPAI viruses identified in wild birds in those areas of Asia (Figure 2), which represent staging areas for wild birds migrating to Europe. However, where this reassortment event occurred cannot be assessed from the available data. Analysis of molecular markers associated with zoonotic potential demonstrated the absence in the HA and polymerase basic protein 2 genes of major signatures associated with increased replication in humans.

## Conclusions

Our data describe a novel HPAI H5N8 genotype of clade 2.3.4.4b in Europe, in which 6 gene segments originated from sub-Saharan Africa HPAI H5N8 clade 2.3.4.4b viruses and 2 gene segments from Eurasia LPAI viruses. It has been shown that Africa might serve as a strong epidemiologic area of circulation for Gs/GD H5 (*10*). However, despite extensive circulation in poultry in several countries in Africa, virus spread by wild birds from Africa to Europe has not been documented.

Possible introduction of an influenza A virus from Africa into Eurasia might be caused by widespread virus circulation in previously unaffected areas, high prevalence of the virus in wild birds in Africa, or alterations of the migratory bird exposure risks after changes in migratory routes caused by unusual weather patterns. Identification of highly related virus strains 1 year apart in 2 countries in Africa >5,000 km apart confirms the high mobility of the HPAI H5N8 virus and suggests a gap in surveillance efforts in these areas. The lack of such surveillance data makes it impossible to determine where this virus originated and whether it spread from South Africa to Nigeria or to both countries from unsampled locations, and to assess its prevalence in Africa.

Our results suggest that H5N8 virus might have been spread from Africa to Asia or Europe by wild bird migratory movements, likely in 2019, although other routes of virus spread cannot be ruled out. Until its detection in Poland in December 2019, the virus might have circulated in an unknown region of Europe or Asia where it reassorted with local LPAI strains of the Eurasian lineage, although we cannot completely exclude that this reassortment event might have occurred in Africa. Late detection in Europe (end of December 2019), compared with the epidemic wave during 2016–17 (October 2016), might be explained by unusually mild temperatures in molting areas in Russia during November and December 2019 (*14**,**15*) and the late westward movement of infected wintering wild birds.

Subclinical infections or insufficient active surveillance efforts in clinically healthy wild population might be the cause of the few detections of the H5N8 virus in wild birds in Eurasia and along the Africa–Eurasia flyways. A better understanding of factors regulating wild bird migrations, as well as increasing wild and domestic bird surveillance in Africa and Eurasia, is needed to improve our ability to early detect and monitor virus spread.

Appendix Additional information on sub-Saharan Africa and Eurasia ancestry of reassortant highly pathogenic avian influenza A(H5N8) virus, Europe, December 2019.
