# Supplementary material for: Sub-Saharan Africa and Eurasia Ancestry of Reassortant Highly Pathogenic Avian Influenza A(H5N8) Virus, Europe, December 2019
Source: Emerg Infect Dis. 2020 Jul;26(7):1557–61. doi: 10.3201/eid2607.200165 (PMC7323556; doi:10.3201/eid2607.200165)
Supplement: Appendix — Additional information on sub-Saharan Africa and Eurasia ancestry of reassortant highly pathogenic avian influenza A(H5N8) virus, Europe, December 2019. [file 20-0165-Techapp-s1.pdf]

Article DOI: https://doi.org.10.3201/eid2607.200165

Sub-Saharan Africa and Eurasia Ancestry of Reassortant Highly Pathogenic Avian Influenza A(H5N8) Virus, Europe, December 2019

Appendix

Genome Amplification and Sequencing of H5N8 Virus from Nigeria

Total RNA was purified from allantoic fluid by using the Viral RNA Kit (QIAGEN, https://www.qiagen.com). The complete genome of the virus from Nigeria was amplified by using the SuperScript III One-Step RT-PCR System and Platinum Taq High Fidelity (Invitrogen, https://www.thermofisher.com) as described (*1*,*2*). The sequencing library was prepared by using the Nextera DNA XT Sample Preparation Kit (Illumina, https://www.illumina.com) and quantified by using the Qubit dsDNA High Sensitivity Kit (Invitrogen). The average fragment length was assessed by using the High Sensitivity DNA Analysis Kit (Agilent Technologies, https://www.agilent.com). The sample was sequenced on an Illumina MiSeq platform, according to the manufacturer's instructions. Read quality was assessed by using FastQC version 0.11.2 (https://www.bioinformatics.babraham.ac.uk).

Raw data were filtered by removal of reads with >10% of undetermined bases, reads with >100 bases with a Q score 80 bases were aligned against a reference genome by using BWA version 0.7.12 (*3*). Potential errors were corrected and base quality was recalibrated by using Picard Tools version 2.1.0 (http://picard.sourceforge.net) and GATK version 3.5 (*4*–*6*). Single-nucleotide polymorphisms were called by using LoFreq version 2.1.2 (*7*). Results were used to generate consensus sequences.

Genome Amplification and Sequencing of H5N8 Virus from Poland

RNA was extracted by using RNeasy Mini Kit (QIAGEN) according to the manufacturer's instruction. Complete genome was obtained by using the One Step RT-PCR Kit (QIAGEN) and specific primers as described (*8*). Nucleotide sequences were determined by using the BigDye Terminator version 3.1 Cycle Sequencing Kit (Applied Biosystems, https://www.thermofisher.com) according to the manufacturer's instruction. Capillary electrophoresis was performed by using a 3500

Genetic Analyzer (Applied Biosystems), and sequences obtained were assembled and analyzed by using SeqMan, DNAStar LaserGene version 8.1.3 (https://www.dnastar.com/software/lasergene).

Phylogenetic Analyses

Consensus sequences of the 8 gene segments of the viruses from Nigeria and Poland were compared with the most related sequences available in GISAID (https://www.gisaid.org) (Appendix Table) and aligned by using MAFFT version 7 (*9*). Maximum-likelihood phylogenetic trees were generated in IQTREE version 1.6 (http://www.iqtree.org/release/v1.6.8) and performing ultrafast bootstrap resampling analysis (1,000 replications) (*10*,*11*). Phylogenetic trees were visualized by using FigTree version 1.4.2 (http://tree.bio.ed.ac.uk).

References

1. Fusaro A, Zecchin B, Vrancken B, Abolnik C, Ademun R, Alassane A, et al. Disentangling the role of Africa in the global spread of H5 highly pathogenic avian influenza. Nat Commun. 2019;10:5310. PubMed https://doi.org/10.1038/s41467-019-13287-y

2. Zhou B, Donnelly ME, Scholes DT, St George K, Hatta M, Kawaoka Y, et al. Single-reaction genomic amplification accelerates sequencing and vaccine production for classical and Swine origin human influenza a viruses. J Virol. 2009;83:10309–13. PubMed https://doi.org/10.1128/JVI.01109-09

3. Li H, Durbin R. Fast and accurate long-read alignment with Burrows-Wheeler transform. Bioinformatics. 2010;26:589–95. PubMed https://doi.org/10.1093/bioinformatics/btp698

4. McKenna A, Hanna M, Banks E, Sivachenko A, Cibulskis K, Kernytsky A, et al. The genome analysis toolkit: a MapReduce framework for analyzing next-generation DNA sequencing data. Genome Res. 2010;20:1297–303. PubMed https://doi.org/10.1101/gr.107524.110

5. DePristo MA, Banks E, Poplin R, Garimella KV, Maguire JR, Hartl C, et al. A framework for variation discovery and genotyping using next-generation DNA sequencing data. Nat Genet. 2011;43:491–8. PubMed https://doi.org/10.1038/ng.806

6. Van der Auwera GA, Carneiro MO, Hartl C, Poplin R, del Angel G, Levy-Moonshine A, et al. From FastQ date to high-confidence variant calls: the genome analysis toolkit best practices pipeline. Current Protocols in Bioinformatics. 2013;43:11.10.1–11.10.33.

7. Wilm A, Aw PP, Bertrand D, Yeo GH, Ong SH, Wong CH, et al. LoFreq: a sequence-quality aware, ultra-sensitive variant caller for uncovering cell-population heterogeneity from high-throughput sequencing datasets. Nucleic Acids Res. 2012;40:11189–201. PubMed https://doi.org/10.1093/nar/gks918

8. Świętoń E, Śmietanka K. Phylogenetic and molecular analysis of highly pathogenic avian influenza H5N8 and H5N5 viruses detected in Poland in 2016–2017. Transbound Emerg Dis. 2018;65:1664–70. PubMed https://doi.org/10.1111/tbed.12924

9. Katoh K, Standley DM. MAFFT multiple sequence alignment software version 7: improvements in performance and usability. Mol Biol Evol. 2013;30:772–80. PubMed https://doi.org/10.1093/molbev/mst010

10. Hoang DT, Chernomor O, von Haeseler A, Minh BQ, Vinh LS, Rosenberg MS. UFBoot2: improving the ultrafast bootstrap approximation. Mol Biol Evol. 2018;35:518–22. PubMed https://doi.org/10.1093/molbev/msx281

11. Nguyen L-T, Schmidt HA, von Haeseler A, Minh BQ. IQ-TREE: a fast and effective stochastic algorithm for estimating maximum-likelihood phylogenies. Mol Biol Evol. 2015;32:268–74. PubMed https://doi.org/10.1093/molbev/msu300

Appendix Table. Information from the GISAID EpiFlu database on hemagglutinin segments used for study of sub-Saharan Africa and Eurasia ancestry of reassortant highly pathogenic avian influenza A(H5N8) virus, Europe, December 2019*

Segment ID	Country	Collection date	Isolate ID	Isolate name	Originating laboratory	Submitting laboratory	Authors
EPI1032508	Hungary	2017 Apr 20	EPI_ISL_271708	A/Goose/Hungary/17985/2017	National Food Chain Safety Office Veterinary Diagnostic Directorate Laboratory for Molecular Biology	Central Agricultural Office Veterinary Diagnostic Directorate	Adam D
EPI1061370	Belgium	2017 Jun 15	EPI_ISL_278200	A/Brahma chicken/Belgium/6153/2017	NA	Public domain	Steensels M, Lambrecht B, Vandenbussche F, Van Borm S.
EPI1144334	United Kingdom	2017 Dec 31	EPI_ISL_292223	A/mute_swan/England/AVP_18_001986/2017	Animal and Plant Health Agency	Animal and Plant Health Agency	Seekings J, Ellis R, Brookes SM, Reid S, Essen S, Lewis N, Brown IH
EPI1144409	China	2016 May 13	EPI_ISL_292235	A/Bar-headed Goose/Qinghai/a43/2016	Wuhan Institute of Virology,	Wuhan Institute of Virology	NA
EPI1145853	China	2016 May 14	EPI_ISL_292496	A/Bar-headed Goose/Qinghai/A11/2016	Wuhan Institute of Virology	Wuhan Institute of Virology	NA
EPI1185001	Poland	2017 Feb 6	EPI_ISL_300703	A/domestic_goose/Poland/124/2017	National Veterinary Research Institut Poland	National Veterinary Research Institut Poland	Swieton E, Smietanka K
EPI1185019	Poland	2017 Mar 8	EPI_ISL_300705	A/turkey/Poland/285/2017	National Veterinary Research Institut Poland	National Veterinary Research Institut Poland	Swieton E, Smietanka K
EPI1185049	Poland	2017 Feb 21	EPI_ISL_300747	A/mute_swan/Poland/137/2017	NA	National Veterinary Research Institut Poland	Swieton E, Smietanka K
EPI1195315	The Netherlands	2018 Feb 26	EPI_ISL_302826	A/Chicken/Netherlands/EMC-14/2018	Erasmus Medical Center	Erasmus Medical Center	Poen MJ, Bestebroer TM, De Meulder D, Vuong O, Scheuer RD; Netherlands Food and Consumer Product Safety Authority, Koopmans MP, Fouchier RA
EPI1208386	The Netherlands	2018 Mar 13	EPI_ISL_305416	A/Domestic_Duck/Netherlands/EMC-2/2018	Erasmus Medical Center	Erasmus Medical Center	Poen MJ, BestebroerTM, De Meulder D, Vuong O, Scheuer RD; Netherlands Food and Consumer Product Safety Authority, Koopmans MP, Fouchier RA
EPI1223834	Democratic Republic of the Congo	2017 May 14	EPI_ISL_308803	A/duck/Democratic Republic of the Congo/17RS882–33/2017	NA	Public domain	Twabela A, Zecchin B, Tshilenge G, Sakoda Y, Kone P, Zamperin G, Drago A, Monne I

EPI1223853	Cameroon	2017 Jan 1	EPI_ISL_308806	A/chicken/Cameroon/17RS1661–1/2017	NA	Public domain	Poen MJ, BestebroerTM, De Meulder D, Vuong O, Scheuer RD; Netherlands Food and Consumer Product Safety Authority, Koopmans MP, Fouchier RA
EPI1223854	Cameroon	2017 Jan 1	EPI_ISL_308807	A/duck/Cameroon/17RS1661–3/2017	NA	Public domain	Poen MJ, BestebroerTM, De Meulder D, Vuong O, Scheuer RD; Netherlands Food and Consumer Product Safety Authority, Koopmans MP, Fouchier RA
EPI1279361	Germany	2018 Aug 31	EPI_ISL_322179	A/domestic duck/Germany-MV/AR613-L02727/2018	NA	Friedrich-Loeffler-Institut	NA
EPI1327144	The Netherlands	2018 Aug 24	EPI_ISL_332441	A/Mallard/Netherlands/18012508–017/2018	Wageningen Bioveterinary Research	Wageningen Bioveterinary Research	Beerens N, Heutink R, Harders F, Verschuren-Pritz S, Bossers A, Koch G, Bergervoet S
EPI1398282	Nigeria	2016 Nov 19	EPI_ISL_348279	A/duck/Nigeria/17RS737–43/2016	National Veterinary Research Institute	Istituto Zooprofilattico Sperimentale Delle Venezie	Joannis T, Meseko C, Zamperin G, Milani A, Zecchin B, Fusaro A, Salviato A, Schivo A, Monne I
EPI1514110	South Africa	2017 Aug 1	EPI_ISL_369339	A/chicken/South Africa/440638A/2017	NA	Public domain	Abolnik C, Rauff DL, Pieterse R, Peyrot B, Choma P, Ebersohn K, Phiri T
EPI1514158	South Africa	2017 Aug 17	EPI_ISL_369345	A/chicken/South Africa/443397/2017	NA	Public domain	Abolnik C, Rauff DL, Pieterse R, Peyrot B, Choma P, Ebersohn K, Phiri T
EPI1514318	South Africa	2017 Sep 29	EPI_ISL_369365	A/chicken/South Africa/451457/2017	NA	Public domain	Abolnik C, Rauff DL, Pieterse R, Peyrot B, Choma P, Ebersohn K, Phiri T
EPI1514326	South Africa	2017 Oct 26	EPI_ISL_369366	A/ostrich/South Africa/002/2017	NA	Public domain	Abolnik C, Rauff DL, Pieterse R, Peyrot B, Choma P, Ebersohn K, Phiri T
EPI1553392	Uganda	2017 Jan 01	EPI_ISL_379227	A/chicken/Uganda/17RS115–15/2017	NA	Public domain	Ndumu D, Zecchin B, Fusaro A, Arinaitwe E, Erechu R, Kidega E, Kayiwa J, Muwanga E, Kirumira M, Kirembe G, Lutwama J, Monne I
EPI1575277	Zimbabwe	2017 May 25	EPI_ISL_387133	A/chicken/Zimbabwe/AI4935/2017	NA	Public domain	Rotherham LS, van Schalkwyk A, Romito M

EPI548623	The Netherlands	2014 Nov 15	EPI_ISL_168075	A/chicken/Netherlands/14015531/2014	Wageningen Bioveterinary Research	Wageningen Bioveterinary Research	Heutink R, Harders R, Verschuren-Pritz S, Bossers A, Koch G, Bouwstra R
EPI858836	India	2016 Oct 17	EPI_ISL_237553	A/duck/India/10CA01/2016	National Institute of High Security Animal Diseases	National Institute of High Security Animal Diseases	Nagarajan S, Tripathi S, Kumar M, Murugkar HV, Tosh C, Singh VP
EPI858844	India	2016 Oct 20	EPI_ISL_237554	A/painted stork/India/10CA03/2016	National Institute of High Security Animal Diseases	National Institute of High Security Animal Diseases	Nagarajan S, Tripathi S, Kumar M, Murugkar HV, Tosh C, Singh VP
EPI869929	Poland	2016 Dec 21	EPI_ISL_240106	A/mute swan/Poland/108/2016	NA	National Veterinary Research Institut Poland	Swieton E, Smietanka K
EPI869931	Poland	2016 Dec 19	EPI_ISL_240108	A/turkey/Poland/83/2016	NA	National Veterinary Research Institut Poland	Swieton E, Smietanka K
EPI926613	Russia	2016 Oct 4	EPI_ISL_240677	A/domestic duck/Siberia/103/2016	Research Institute of Experimental and Clinical Medicine	Research Institute of Experimental and Clinical Medicine	Sharshov KA, Kurskaya OG, Alexeev AY, Sobolev IA, Alikina TY, Kabilov MR, Shestopalov AM
EPI954567	Italy	2017 Jan 20	EPI_ISL_255182	A/turkey/Italy/17VIR538–1/2017	Istituto Zooprofilattico Sperimentale Delle Venezie	Istituto Zooprofilattico Sperimentale Delle Venezie	Zecchin B, Fusaro A, Milani A, Schivo A, Salviato A, Zamperin G, Marciano S, Ormelli S, Terregino C, Monne I

*All submitters of data can be contacted directly through the GISAID website (https://www.gisaid.org). NA, not available.
